# Iron reduction via periodic calibrated phlebotomy to target ferritin lowers cancer incidence and mortality in patients with peripheral artery disease: further insights from the FeAST trial

**DOI:** 10.3389/fonc.2025.1695261

**Published:** 2025-11-26

**Authors:** Paul Pisarik

**Affiliations:** Mercy Health, Oklahoma City, OK, United States

**Keywords:** phlebotomy methods, ferritins, blood, neoplasms, prevention and control, mortality, risk reduction behavior, statistics and numerical data

## Introduction

The prospectively collected cancer substudy data within the Iron in Atherosclerosis Study (FeAST), led by the late Dr. Leo Zacharski, highlights a previously unrecognized substantial therapeutic potential of iron reduction in lowering cancer incidence and mortality (1). This study demonstrated that iron reduction via calibrated phlebotomy, targeting a ferritin level of 25 ng/mL, significantly decreased cancer risk in the general study population and cancer-specific mortality and all-cause mortality in those diagnosed with cancer in a Veteran Affairs Peripheral Arterial Disease (PAD) population (mean age, 67 years: 98.5% self-reported as male). Notably, these effects first became significant within 6 months of the initial series of phlebotomies. An additional analysis shows how powerful this effect is.

## Analysis

The number needed to treat (NNT) was calculated for two of the main findings of the study. The study reported a mean achieved ferritin level of 79.7 ng/mL (down from a mean of 121.8 ng/mL at study onset in the actively treated group), representing an average blood withdrawal of 411 mL twice yearly. With two-sided tests of statistical significance and the statistical significance set at 0.05, the key findings included the following:

1. Reduced incidence of new cancer diagnoses (HR = 0.65; 95% CI = 0.43–0.97, *p* = 0.036) 38/636 (6.0%) vs. 60/641 (9.3%), NNT = 29.5 over 4.5 years.
2. Lower cancer-specific mortality (HR = 0.39; 95% CI = 0.21–0.72, *p* = 0.003) 14/636 (2.2%) vs. 36/641 (5.6%), NNT = 29.3 over 4.5 years.
3. Decreased all-cause mortality in those diagnosed with cancer (HR = 0.49; 95% CI = 0.29–0.83, *p* = 0.009). Numbers to calculate NNT not given in the paper.

To place these findings in context, the NNT over a 10-year period was calculated by using calibrated phlebotomy, assuming a sustained linear benefit from iron reduction.

- To prevent any cancer death, NNT = 13, which means that in a cohort of 1,000 individuals over 10 years, 49 who had cancer would not die of it.
- To prevent any cancer, NNT = 13, which means that another 49 individuals in a cohort of 1,000 individuals over 10 years would avoid developing cancer.

The toxic effect of ferritin is also reflected in the observation that as the study population got older, the baseline ferritins of those older individuals got lower, consistent with the healthy survivor effect (2).

## Discussion

Therefore, in a cohort of 1,000 subjects, predominantly male, with PAD, semiannual phlebotomy to maintain a ferritin level of ~80 ng/mL over 10 years would prevent 98 individuals from dying of cancer if diagnosed with cancer or developing cancer in the first place (NNT = 7). Although not directly comparable with other cancer screening measures owing to their being in a heterogenous population and hence being at a probable lower risk, the NNT for phlebotomy for preventing cancer and cancer-related deaths in this male cohort is 140 times more effective than prostate cancer screening is in preventing prostate cancer deaths over 10 years in men aged 55–69 (number needed to screen = 1,000) (3).

LabCorp has a normal range of ferritin being 30–400 ng/mL in male patients and 13–150 ng/mL for female patients (4), while Quest Diagnostics has 24–380 ng/mL as a normal for older male patients and 16–288 ng/mL as a normal range for older female patients (5). In this study, just dropping the average from 121.8 to 79.7 ng/mL dramatically lowered the cancer incidence and mortality rates. Much like the outdated assumption that blood pressures up to 160/90 were once considered “normal” for older adults, the late Dr. Ralph DiPalma suggested the new normal range for ferritin as 20–100 ng/mL (6).

## Conclusions

Based on the FeAST study,

1. Men with PAD, without a history of visceral cancer, should donate blood, whenever possible, to greatly lessen their chances of getting cancer or dying from it.

2. This benefit for the general population, at this point, can only be a theoretical extrapolation. In addition, in *post-hoc* analyses, smoking tobacco was of borderline significance for being an effect modifier for total mortality, and since smoking tobacco has decreased since the study was done, the results even in the narrow VA population targeted earlier could have been different had the study been done today (7).

Future studies targeting iron reduction to reduce cancer incidence and mortality in the general population should take into account the findings of the FeAST study to target the “right” heterogeneous population to achieve the maximal potential benefits of iron reduction.

3. If future studies confirm the FeAST study results, there needs to be an updated and narrowed normal range for ferritin.
